# Prevalence of Adolescent Problem Gambling: A Systematic Review of Recent Research

**DOI:** 10.1007/s10899-016-9627-5

**Published:** 2016-07-02

**Authors:** Filipa Calado, Joana Alexandre, Mark D. Griffiths

**Affiliations:** 1 International Gaming Research Unit, Psychology Division, Nottingham Trent University, Burton Street, Nottingham, NG1 4BU United Kingdom; 2 ISCTE – Lisbon University Institute, Avenida das Forças Armadas, 1649-026 Lisbon, Portugal

**Keywords:** Adolescence, Gambling, Problem gambling, Gambling prevalence, Adolescent gambling, Youth gambling

## Abstract

Previous research has shown that gambling is a popular activity among adolescents. Following a rapid expansion of legalized gambling opportunities and the emergence of new forms of gambling, many researchers have carried out studies on adolescent gambling and problem gambling. The present paper reviews studies that have been conducted worldwide since 2000, and then presents a more detailed picture of adolescent gambling research in Europe, by providing a country-by country analysis. After an extensive search on academic databases and following an exclusion process, 44 studies were identified. The findings showed that 0.2–12.3 % of youth meet criteria for problem gambling, notwithstanding differences among assessment instruments, cut-offs, and timeframes. However, despite this variability, several demographic characteristics were associated with adolescent gambling involvement and problem gambling. It is concluded that a small but significant minority of adolescents have gambling-related problems. Such findings will hopefully encourage more research into youth gambling to further understand the determinants of this phenomenon.

## Introduction

International studies have consistently shown that gambling is part of the life experiences of most young people (Hayer and Griffiths 2014). Furthermore, the current generation of youth have grown up in an era where gambling opportunities are widespread (Volberg et al. 2010; Gupta and Derevensky 2000). In addition, the development of technology has generated new forms of gambling via the Internet, mobile phone and interactive television (Griffiths and Parke 2010). It has also been argued that youth are receptive to modern forms of gambling because of the apparent similarity between these games and other familiar technology-based games (Delfabbro et al. 2009).

Furthermore, the increased availability of legal gambling appears to have led to some increases in the prevalence of adolescent gambling and to the development of gambling problems among young people. Recent studies (e.g., Molinaro et al. 2014) indicate that in spite of adolescent gambling being an illegal activity, youth engage in gambling with a prevalence rate higher than adults (Gupta and Derevensky 2000; Volberg et al. 2010).

Thus, concerns about adolescent gambling have encouraged public health workers to study the epidemiology of gambling as this helps to characterize this phenomenon (Gupta and Derevensky 2014). Consequently, there is a need for conducting a systematic review in order to synthesize the trends in adolescent gambling and to analyse the comparative prevalence of problem gambling rates across different countries.

Therefore, the aim of the present review is twofold. Firstly, to briefly review the most recent international research published since 2000, with respect to problem gambling prevalence rates among adolescents, as the past few decades have witnessed an unprecedented growth in the gambling industry, which could have led to the development of gambling-related problems among young people (Meyer et al. 2009). Secondly to present a more detailed picture of adolescent gambling in Europe. Although there are other reviews in the literature concerning adolescent gambling (e.g., Volberg et al. 2010; Kristiansen and Jensen 2011), these are now outdated and did not provide a country-by-country overview of adolescent gambling across a whole continent. Therefore, the present review updates and expands on previous reviews and provides a brief country-by-country analysis of the evidence of adolescent gambling and problem gambling in that particular country in alphabetical order.

## Methods

A literature search was carried out using the following databases: Scopus, PsycINFO, Science Direct, PsycARTICLES, PubMED, Wiley Online Library, ProQuest Dissertations and Theses Academic Search complete and Google Scholar. The following search terms were used: “youth gambling prevalence” “adolescent gambling” “adolescent problem gambling” “youth gambling addiction” “youth compulsive gambling”. The search was conducted with the same terms in English, French, Spanish and Portuguese, in order to obtain as many prevalence studies as possible and to avoid English publication bias.

The studies were selected on the basis of containing the following criteria: (1) being published since 2000; and (2) citing gambling prevalence rates for adolescents and young people (with an age that could range from 10 to 24 years). Moreover, reference lists of retrieved studies and from other reviews already available in the literature were also searched in order to identify any additional relevant studies. The goal was to locate all prevalence studies that were conducted at a national level. Therefore, for countries that had prevalence data at both regional and national level, only national data were considered. However, in the case of countries that did not have a national prevalence study, but instead had conducted studies at a regional level with a representative sample, these studies were included. Studies were also excluded if they (1) had a sample size of less than 500 participants, (2) did not use a standardized instrument to assess problem gambling, and (3) assessed problem gambling in the context of a specific form of gambling, such as Internet gambling.

## Results

In the first step, 54 studies were identified after a careful examination of the titles and abstracts of the studies generated by the search on the aforementioned databases and through reference lists from other studies and reviews. In the second step, studies were excluded due to the following criteria: (1) a sample of less than 500 participants (three studies); (2) did not use a standardized measure to assess problem gambling (five studies), and (3) only examined gambling and problem gambling in the context of a specific form of gambling (two studies). Therefore, the final search yielded 44 studies, which are summarized in Table 1. The majority of studies were published in English (n = 42), with one study in French, and one study in Spanish. Two studies were conducted in North America, one in South America, one in Asia, five in Oceania, and 35 in Europe.

**Table 1:** Overview of adolescent gambling prevalence studies across the world

Country	Study	Measure	Sample characteristics	Response rate	Gambling prevalence	Problem gambling prevalence	Legal age to gamble
*North America*							
Canada	Huang and Boyer (2007)	CPGI	National, 5666 youth aged 15- 24 years that completed a survey using computer assisting interviews	77 %	61.35 % (past year)	CPGI: Problem gambling (3+): 2.2 % (past-year prevalence)	18 years for Alberta, Manitoba, Quebec and 19 years for other states
USA	Welte et al. (2008)	SOGS-RA and DIS	National, 2274 youth aged 14–21 years, who were surveyed by telephone	Not reported	68 % (past year prevalence)	SOGS-RA: Problem gambling (4+): 2.1 % (past-year prevalence) DIS: Problem gambling (+3): 2.2 %, pathological (+4):0.4 %; Combined rate: 2.6 % (past-year prevalence)	From 12 to 21 years depending on states and gambling activities
*South America*							
Brazil	Spritzer et al. (2011)	DSM-IV-J	661 with adolescents aged 14–17 years (these participants were a sub-sample of the general population), who were interviewed face-to-face	66.4 and 81 % for the adolescent sub-sample	6.9 % (no specific time frame is provided)	DSM-IV-J Problem gambling (+2): 1.6 % (lifetime prevalence)	18 years
*Asia*							
Hong Kong	Hsu et al. (2014)	DSM-IV-MR-J	926 youth aged 12–20 years, who completed the survey in the class	84.5 %	46.5 % (past-year)	DSM-IV-MR-J: Problem gambling (4+): 0.9 % (past-year prevalence)	18 years
*Oceania*							
Australia	Delfabbro and Thrupp (2003)	DSM-IV-J	South Australia, 505 youth aged 15–17, who completed the survey in the class	Not reported	47.8 % (past-year)	DSM-IV-J: Problem gambling (4+): 3.5 % (past-year prevalence)	18 years for most of gambling activities and 16 years for lotteries in some states
	Delfabbro et al. (2005)	DSM-IV-J and VGS	ACT region, 926 young people aged 11–19 years, who completed a survey in the class	Not reported	70.4 % (past-year)	DSM-IV-J: Problem gambling (4+): 4.4 % (past-year prevalence) VGS: Problem gambling (21+): 3.3 % (no information regarding time frame is provided)	
	Lambos et al. (2007)^a^	DSM-IV-J	South Australia, 2669 young people aged 13–17 years, who completed the survey in the class	Not reported	56.3 % (past-year)	DSM-IV-J: Problem gambling (4+): 2.4 % (past-year prevalence)	
	Delfabbro and King (2011)	DSM-IV-J	Darwin metropolitan area, 1107 aged 14–18 years, who completed the survey in the class	Not reported	50.8 % (past-year prevalence for participants aged less than 18 years of age)	DSM-IV-J: Problem gambling (4+): 0.2 % (past-year prevalence)	
New Zealand	Rossen (2008)^a^	DSM-IV-MR-J	2005 youth aged 11–21 years in the Upper North Island, who completed the survey in the class	Not reported	65.4 % (past-year)	DSM-IV-MR-J: Problem gambling (4+): 3.8 % (past- year prevalence)	18 years for most gambling activities and 20 years to enter a casino
*Europe*							
Albania	Molinaro et al. (2014)	Lie/Bet	National data from ESPAD, 3189 students aged 16 years, who completed the survey in the class	90 %	Not reported	Lie/Bet: Probable problem gambling (sore of 2): 5.3 % (lifetime prevalence)	18 years
Croatia	Dodig (2013)	CAGI	1948 students aged 14–20 years from Zagreb, Osijek, Rijeka, and Split, who completed the survey in the class	Not reported	Not reported	CAGI: Problem gambling (6+): 12.3 % (past three months prevalence)	18 years
Cyprus	Molinaro et al. (2014)	Lie/Bet	National data from ESPAD, 4243 students aged 16 years, who completed the survey in the class	Not reported^b^	Not reported	Lie/Bet: Probable problematic gambling (score 2): 4.4 % (lifetime prevalence)	18 years
Denmark	Kristiansen and Jensen (2014)	SOGS-RA	National, 2223 adolescents aged 11–17 years, who completed the survey in the class	91 %	70.1 % (past-year)	SOGS-RA: Problem gambling: 1.3 % (past-year prevalence)	18 years for casinos, slot machines and Internet, and 16 years for other kinds of gambling
	Molinaro et al. (2014)	Lie/Bet	National data from ESPAD, 2181 students aged 16 years, who completed the survey in the class	89 %	Not reported	Lie/Bet: Probable problem gambling (score 2): 1.6 % (lifetime prevalence)	
Finland	Ilkas and Aho (2006)	SOGS-RA	5000 adolescents aged 12–17 years	Not reported^c^	52 % (past-year)	SOGS-RA: Problem gambling (5+):1.3 % (past-year prevalence)	18 years
	Molinaro et al. (2014)	Lie/Bet	National data from ESPAD, 3744 students aged 16 years, who completed the survey in the class	90 %	Not reported	Lie/Bet: Probable problem gambling (score 2): 4.8 % (lifetime prevalence)	
Germany	Hurrelmann et al. (2003)	DSM-IV-MR-J	Regional, 5009 adolescents aged 13–19 years from Federal State of North Rhine–Westphalia	^d^	39.9 % (past-year)	DSM-IV-MR-J: Problem gambling: 2.96 % (past-year prevalence)	18 years
	Duven et al. (2011); Hayer (2014)	DSM-IV-MR-J	Regional, 3967 students aged 12–18 years from Rhineland-Palatinate	^d^	41.2 % (past year)	DSM-IV-MR-J: Problem gambling: 2.2 % (past-year prevalence)	
Great Britain	Ashworth and Doyle (2000)^a^	DSM-IV-MR-J	9529 adolescents aged 12–15 years from England and Wales, who completed the survey in the class	40 % (school response rate)	34 % (past seven days)	DSM-IV-MR-J: Problem gambling (4+): 5.4 % (past-year prevalence)	16 years for lotteries and scratchcards and 18 years for other gambling activities
	Griffiths and Wood (2007); Griffiths (2008)	DSM-IV-MR-J	National, 8017 adolescents aged 12–15 years, who completed the survey in the class	26 % (school response rate)	73 % (lifetime)	DSM-IV-MR-J: Problem gambling (4+): 3.5 % (past-year prevalence)	
	Forrest and McHale (2012)	DSM-IV-MR-J	National, 8958 aged 11–15 years, who completed the survey in the class	22 % (school response rate)	21 % (past seven days)	DSM-IV-MR-J: Problem gambling (4+): 1.9 % (past-year prevalence)	
	Ipsos MORI (2014)^a^	DSM-IV-MR-J	2796 students aged 11–16 years from England and Wales, who completed the survey in the class	20 % (school response rate)	16 % (past seven days among people aged 11–15 years)	DSM-IV-MR-J: Problem gambling (4+): 0.8 % (past-year prevalence)	
United Kingdom	Molinaro et al. (2014)	DSM-IV-MR-J	National data from ESPAD, 1712 students aged 16 years, who completed the survey in the class	81 %	Not reported	Lie/Bet: Probable problem gambling (score 2): 2.2 % (lifetime prevalence)	
Iceland	Olasson et al. (2006a)	SOGS-RA and DSM-IV-MR-J	Reykjavik and Akureyri region, 750 adolescents aged 16–18 years, who completed the survey in the class	Not reported	79.1 % (past-year)	DSM-IV-MR-J: Problem gambling (4+): 2 % SOGS-RA: Problem gambling (4+): 2.7 % (past-year prevalence)	18 years
	Olasson et al. (2006b)	SOGS-RA and DSM-IV-MR-J	Reykjavík region, 3511 adolescents aged 13–15 years, who completed the survey in the class	84 %	70 % (past-year)	DSM-IV-MR-J: Problem gambling (4+):1.9 % SOGS-RA: Problem gambling (4+): 2.8 % (past-year prevalence rates)	
	Olason et al. (2011)	DSM-IV-MR-J	Hafnarfjörður region, 1537 adolescents aged 13–18 years, who completed the survey in the class	81.4 %	56.6 % (past-year)	DSM-IV-MR-J: Problem gambling (4+): 2.2 % (past-year prevalence)	
Italy	Bastiani et al. (2011)	CPGI-short form	National, 1241 youth aged 15–24 years derived from the ISPAD survey, who completed an anonymous postal survey	35 %	35.7 % (past-year)	CPGI: Problem gambling (3+): 2.3 % (past-year prevalence)	18 years
	Molinaro et al. (2014)	Lie/Bet	National data from ESPAD, 4837 students aged 16 years, who completed the survey in the class	Not reported^b^	Not reported	Lie/Bet; Probable problem gambling (score 2): 2.6 % (lifetime prevalence)	
Lithuania	Skokauskas and Satkeviciute (2007)	SOGS-RA and DSM-IV-MR-J	Kaunas city, 835 youth aged 10–18 years, who completed the survey in the class	96 %	82.7 % (lifetime)	SOGS-RA: Problem gambling: 5.2 % (past-year prevalence) DSM-IV-MR-J: Problem gambling (4+): 4.2 % (past-year prevalence)	21 years for casino operated games and 18 years for other gambling activities. There is no legal prohibition for lotteries
	Molinaro et al. (2014)	Lie/Bet	National data from ESPAD, 2476 students aged 16 years, who completed the survey in the class	89 %	Not reported	Lie/Bet: Probable problem gambling (score 2): 4.2 % (lifetime prevalence)	
Norway	Johansson and Götestam (2003)	DSM-IV	National, 3237 adolescents aged 12–18 years, who completed a telephone and postal interview	45.2 %	24.9 % (past seven days)	DSM-IV: Problem gambling (+3): 3.46 %; pathological gambling (+5): 1.76 %; Combined rate: 5.2 % (lifetime prevalence)	18 years
	Molde et al. (2009)	DSM-IV subscale of Massachusetts Adolescent Gambling Screen (MAGS)	1351 students aged 16–19 years from West Norway, who completed an online survey	69.8 %	Not reported	DSM-IV: Problem gambling (3- 4.5): 1.9 % Pathological gambling (5+): 2.5 % Combined rate: 4.4 % (past-year prevalence)	
	Rossow and Molde (2006)	Lie/Bet and SOGS-RA	National, 20,703 students aged 13 to 19 years, who completed the survey in the class	80.2 %	74.4 % (past-year)	SOGS-RA: Problem gambling: (3–4): 2.5 % (past -year prevalence) Lie/Bet: Problem gambling (2 items): 3.5 % (lifetime prevalence)	
	Rossow et al. (2013) (study conducted before the ban of slot machines in the country)	SOGS-RA and Lie/Bet	National, 4912 adolescents aged 13–18 years, who completed the survey in the class	85.7 %	69.3 % (past-year)	SOGS-RA: Problem gambling (4+): 2.3 % (past-year prevalence) Lie/Bet: Problem gambling (2): 3 % (lifetime prevalence)	
	Rossow et al. (2013) (study conducted after the ban of slot machines in the country)	SOGS-RA and Lie/Bet	National, 3855 adolescents aged 13–18 years, who completed the survey in the class	77.7 %	67 % (past-year)	SOGS-RA: Problem gambling (4+): 3.1 % (past-year prevalence) Lie/Bet: Problem gambling (2): 3.4 % (lifetime prevalence)	
	Hanss et al. (2014)	PGSI	National, 2.059 adolescents aged 17 years, who completed a postal survey	70.4 %	26.1 % (past-month)	PGSI: Problem gambling (8+): 0.2 % (past-year prevalence)	
Romania	Lupu et al. (2002)	GA-20	Cluj, Salaj, and Bacau regions, 500 adolescents aged 14–19 years, who completed the survey in the class	^e^	82 (lifetime)	GA-20: Problem gambling (7+): 6.8 % (lifetime prevalence)	18 years
	Lupu and Todirita (2013)	GA-20	Cluj-Napoca and Harghita counties, 1032 adolescents aged 11–19 years, who completed the survey in the class	Not reported	Not reported	GA 20: Pathological gambling (7+): 3.48 % (lifetime prevalence)	
	Molinaro et al. (2014)	Lie/Bet	National data from ESPAD, 2770 students aged 16 years, who completed the survey in the class	Not reported^b^	Not reported	Lie/Bet: Probable problem gambling (score 2): 4.9 % (lifetime prevalence)	
Serbia	Molinaro et al. (2014)	Lie/Bet	National data from ESPAD survey, 6084 students aged 16 years, who completed the survey in the class	86 %	Not reported	Lie/Bet: Probable problem gambling (score 2): 3.1 % (lifetime prevalence)	18 years
Spain	Becoña et al. (2001)	SOGS-RA	Galicia region, 2790 youth aged 14–21 years, who completed a survey in the class	Not reported	Not reported	SOGS-RA: Problem gambling (4+): 5.6 % (past-year prevalence)	18 years
	Míguez and Becoña (2015)	SOGS-RA	Galicia region, 1447 youth aged 11–16 years, who completed a survey in the class	92.4 %	Not reported	SOGS-RA: Problem gambling (4+): 4.6 % (past-year prevalence)	
Sweden	Fröberg et al. (2015)	PGSI	National, 2570 youth aged 16–24 years, who completed a telephone and postal survey	72.5 %	Not reported	PGSI: Problem gambling (3–27): 4.2 % (past-year prevalence)	18 years
Switzerland	Surís et al. (2011)	SOGS-RA	Canton of Neuchatel, 1126 students aged 15–20 years who completed an online survey	28.4 %	37.5 % (past-year)	SOGS-RA: Problem gambling (4+): 1.3 % (past-year prevalence)	18 years

^a^Non-peer-reviewed papers

^b^These studies were taken from the paper by Molinaro et al. (2014). This paper summarized probable problem gambling across nine European countries using data from ESPAD, and which did not indicate the response rates for each country. After analysing the ESPAD report in detail, it can be observed that student response rates were not available for Cyprus, Italy and Romania, and thus the response rates for these countries were not reported in the present review

^c^This study is published only in Finish. The information reported is based on a English abstract and in the review of Kristiansen and Jensen (2011) conducted among the Nordic countries, and thus it was not possible to obtain more specific information about the methodology, such as the modality of survey used and response rates

^d^These papers are only published in German and the information reported was based on Hayer (2014), and thus it was not possible to obtain more specific information concerning methodology, such as the modality of survey used and the response rates

^e^This paper was not fully available in academic databases and the information reported is based on its abstract, which did not contain more specific information about methodology, such as response rates

Assessment of adolescent problem and pathological gambling used the South Oaks Gambling Screen Revised for Adolescents (SOGS-RA) (Winters et al. 1993), Diagnostic and Statistical Manual-IV adapted format for Juveniles (DSM-IV-J) (Fisher 1992), Canadian Problem Gambling Index (CPGI) (Ferris and Wynne 2001), Gamblers Anonymous Twenty Questions (GA20) (Gamblers Anonymous 1984), Lie/Bet Scale (Johnson et al. 1997), and Canadian Adolescent Gambling Inventory (CAGI) (Tremblay et al. 2010).

The present review considers the combined rate of problem and pathological gambling, as many studies merge problem gambling with pathological gambling compared to behaviour from non-gamblers and non-problem gamblers (e.g., Welte et al. 2008).

### Adolescent Gambling and Problem Gambling Worldwide

The present overview of research on adolescent gambling and problem gambling across the world conducted since 2000 shows that there are many countries that have never carried out studies on adolescent gambling behaviour. In fact, most research on adolescent gambling has been conducted in Europe, North America and Australia. However, despite the lack of research in some countries, studies show that 0.2–12.3 % of youth meet diagnostic criteria for problem gambling across five continents (notwithstanding differences in cut-offs and timeframes among assessment instruments). It should also be noted that there are some variations in problem gambling prevalence rates that occur among different continents: in North America problem gambling prevalence rates ranged from 2.1 to 2.6 %, whereas in Oceania these rates ranged from 0.2 to 4.4 %. In Europe, problem gambling prevalence rates ranged from 0.2 to 12.3 %. Consequently, European studies showed the highest and the lowest adolescent problem gambling prevalence rate. Therefore, in the next section, a more detailed overview about adolescent gambling and problem gambling in the European continent will be presented.

### Adolescent Gambling and Problem Gambling in Europe

The European Union appears to be moving towards a more continued expansion of gambling characterized by the legalization and liberalization of gambling markets over the past few decades (Kingma 2008). This may put more young people at risk of developing gambling-related problems, especially underage youth, and suggests the need to provide a full overview of the European continent.

Since it was proposed to provide a more detailed examination about gambling and problem gambling in each European country, the present review also includes some studies that were not included in the initial overview, such as studies that did not use a standardized measure to assess problem gambling (five studies) or studies that assessed problem gambling in the context of a specific mode of gambling, such as online gambling (two studies). Nonetheless, the criterion for excluding studies with less than 500 participants was maintained. In order to present a more in-depth analysis for each European country, other data from other studies such as a description about the most popular gambling activities, and demographics associated with problem gambling are included. However, there are 22 European countries where no empirical research into adolescent gambling and problem gambling has been carried out: Armenia, Andorra, Austria, Belarus, Bulgaria, Czech Republic, Estonia, France, Hungary, Ireland, Latvia, Liechtenstein, Macedonia, Montenegro, Monaco, The Netherlands, Poland, Portugal, Russia, Slovak Republic, Slovenia and Ukraine.

#### Albania

There are some data available for this country, as a part of a wider study that examined adolescent problem gambling across nine European countries (Molinaro et al. 2014), using data from the 2011 European School Survey Project on Alcohol and Other Drugs (ESPAD) study. The Albanian sample comprised 3189 students aged 16 years. Molinaro et al. (2014) reported a probable problem gambling rate of 5.3 % among Albanian youth using the Lie/Bet Scale.

#### Belgium

As a part of a wider study of youth habits, Kinable (2006) conducted a study with 38,357 adolescents aged 12–18-years. Results showed that 40.1 % of the respondents had gambled during their lifetime on at least one of those gambling activities. During the previous six years, participation rates on those gambling activities had been decreasing each year from 52.5 % in 2001 to 42.2 % in 2005. However, no information was provided with regard to problem gambling prevalence rates.

#### Bosnia and Herzegovina

A survey was conducted among secondary school students in the municipalities of Zenica and Kakanj, which included questions about the frequency of playing games of chance among students and their parents (Softić et al. 2013). No standardized instrument to assess problem gambling was applied to students. From the total of 2370 participants, 6.9 % reported that they often played games of chance, 35.4 % claimed that they played them occasionally, and 57.7 % never played any game. With respect to the type of gambling they had participated in, 29.2 % of the students were involved in betting, 7.4 % played lottery, and 0.9 % played cards.

#### Cyprus

There are some data concerning adolescent gambling in Cyprus, as a part of a wider study that examined adolescent problem gambling across nine European countries (Molinaro et al. 2014), using data from the 2011 ESPAD study. The sample size of Cyprus was 4243 students aged 16 years and the prevalence of probable problem gambling was 4.4 % using the Lie/Bet Scale (Molinaro et al. 2014).

Another more recent study examined online gambling in Cyprus (Floros et al. 2015), using a sample of 2684 adolescent students aged 12–16 years. The students completed a survey that included questions on demographics, the DSM-IV-MR-J, Young’s diagnostic questionnaire (YDQ), the Parental Bonding Instrument (PBI) and the Strengths and Difficulties Questionnaire (SDQ). The results indicated that 19.1 % of the students reported having had some experience using the Internet to gamble online during the past 3 months. Moreover, 2.5 % of the total sample and 13.8 % of those who had gambling experience were considered addicted online gamblers as classified by the DSM-IV-MR-J. Online gambling was associated with significantly higher Internet Addiction scores, lower parental care, and higher parental overprotection. Individuals with serious conduct problems (as assessed using the SDQ questionnaire) were more likely to gamble.

#### Croatia

There is one Croatian study on adolescent gambling conducted by Dodig (2013), which used the CAGI, an instrument created specifically for assessing adolescent problem gambling (Tremblay et al. 2010). The sample comprised 1948 students aged between 14 and 20 years, from Zagreb, Osijek, Rijeka and Split. The results showed that 70.8 % of the participants gambled socially with no gambling-related problems, 16.9 % scored as low to moderate problematic gamblers, and 12.3 % satisfied the criteria for severe gambling-related problems. The findings showed that the proportion of gambling-related problems were more prevalent among young men. Significant predictors of gambling problems included the experience of winning a large amount of money, continuation of gambling after winning money, frequency of gambling, and specific motivations for gambling (“gambling for making me feel better”, “gambling to be better at gambling”, and “gambling for earning money”).

#### Denmark

A national survey on gambling behaviour among Danish school adolescents aged 11–17 years was conducted by Kristiansen and Jensen (2014). The instrument used to assess problem gambling was the SOGS-RA. The overall prevalence rate of problem gambling was 1.3 and a further 4.5 % were considered at-risk gamblers. Older respondents reported more gambling problems, although this relationship did not reach statistical significance (Kristiansen and Jensen 2014). Problem gamblers participated in more different gambling activities than at-risk gamblers, who in turn participated in more activities than non-problem gamblers. Moreover, at-risk gamblers and problem gamblers played via their mobile phones and on the Internet to a greater extent than non-problem gamblers. Furthermore, the most frequently reported reasons for engaging in gambling behaviours were to win money (47.6 %), followed by fun/entertainment (45.8 %), socialising with family and friends (41.8 %), excitement (38.5 %) and curiosity (26 %). Gambling to escape problems and the inability to resist temptation were reported more frequently among the at-risk and problem gamblers than among the non-problem gamblers. It was also reported that problem and at-risk gamblers more often played with friends or alone than with parents.

Adolescent gambling in Denmark was also examined in a study using data from the 2011 ESPAD study (Molinaro et al. 2014). The Danish sample comprised 2181 students aged 16 years and the rate of probable problematic gambling was 1.6 % using the Lie/Bet Scale.

#### Finland

With regard to youth gambling, a study was conducted on behalf of the Finnish Ministry of Social Affairs and Health during 2006 (Ilkas and Aho 2006). A total of 5000 12–17-year-old youths were interviewed and gambling was assessed using the SOGR-RA. Moreover, it was estimated that the risk group for problem gambling included approximately 1.3 % of all youths.

A more recent study was carried out by Raisamo et al. (2013), as a part of the 2011 nationwide Adolescent Health and Lifestyle Survey (AHLS). The gambling questions focused on gambling behaviour and gambling-related harms experienced due to gambling, such as “conflicts with parents”, and “disruptions in school/work”. However, the survey did not use any standardised instrument to assess problem gambling. The sample comprised 4566 adolescents aged 12–18 years old. This study reported that 44 % of the sample had gambled during the past 6 months. Of the adolescents in the sample, 32 % were identified as occasional gamblers (gambled a couple of times a month or less often), and 12 % as frequent gamblers (gambled weekly and daily). In addition, the total number of harms experienced by adolescents during the past 6-month period as a consequence of their gambling was 2.1, and the corresponding rate for occasional gamblers was 1.9. The prevalence of gambling was significantly higher among boys than girls.

Another representative study of Finnish adolescent gambling behaviour was derived from the 2011 ESPAD study (Molinaro et al. 2014). The Finnish sample comprised 3744 participants and the prevalence of probable problematic gambling using the Lie/Bet Scale was 4.8 %.

#### Germany

There are a number of empirical studies examining adolescent gambling in Germany (see Hayer 2014), but the majority of them are only available in the German language. The first study was conducted by Hurrelmann et al. (2003) and used the DSM-IV-MR-J to assess problem gambling. The findings showed that 3 % of the sample were classed as problem gamblers.

A more recent study was conducted by Duven et al. (2011) that comprised 3967 students aged between 12 and 18 years. The problem gambling rate was 2.2 % using the DSM-IV-MR-J (see Hayer 2014).

#### Great Britain

The first study published since 2000 was conducted between June and July 1999, using a representative sample of 9529 students aged 12–15 years (Ashworth and Doyle 2000). The findings of this study showed that 47 % of the students reported that they had ever gambled on National Lottery scratchcards, 40 % had gambled on the National Lottery Draw and 68 % had gambled on fruit machines. Moreover, 5.4 % of young people surveyed were classified as problem gamblers according to the DSM-IV-MR-J. Problem gamblers were more likely to be boys than girls (Ashworth and Doyle 2000).

Another adolescent survey was carried out in 2006 by the Ipsos Mori, a leading market research company in the UK with the International Gaming Research Unit (Griffiths 2008; Griffiths and Wood 2007) using a representative sample of 8017 adolescents aged 12 to 15 years of age. The most popular gambling activities were fruit machines (54 %), private bets (37 %) and scratchcards (28 %). The 2006 survey showed a prevalence of problem gambling of 3.5 % (Griffiths and Wood 2007). The results showed that problem gamblers were more likely to exhibit other potentially addictive behaviours (i.e., to have smoked cigarettes, drunk alcohol and taken illegal drugs in the past week).

Another adolescent survey, comprising 8958 adolescents aged 11–15 years surveyed during late 2008 and 2009, found a problem gambling rate of 1.9 %, as assessed by the DSM-IV-MR-J (Forrest and McHale 2012). According to the 2009 survey, higher problem rates were found for smokers (6.5 %), Asians (3.0 %) and slot machine players (9.7 %). The most popular gambling activity was slot machines, with 9 % of the sample reporting playing them (Forrest and McHale 2012).

More recently, another study was carried out by the Ipsos MORI with 2796 students aged between 11 to 16 years from England and Wales (Ipsos MORI 2014). However, the report focused on students aged 11–15 years in order to better understand underage gambling behaviour. The results indicated that 16 % of students aged 11–15 years reported having gambled in the seven days prior to the survey. The most common forms of gambling were fruit machines (6 %), placing a private bet (5 %) or playing cards for money with friends (4 %) and scratchcards (4 %). The proportion of young people who had played *any* gambling game in the past week was higher among 16 year-olds than all other ages. The findings showed that 0.8 % of all participants were identified as problem gamblers, using the DSM-IV-MR-J. For students aged 12–15 years, 0.7 % were identified as problem gamblers, 1.2 % as ‘at risk’ gamblers, and 12.0 % as social gamblers. Boys were more likely than girls to be classified as problem gamblers (1.1 and 0.2 % respectively). In fact, these results showed a decrease in the number of problem gamblers, in comparison with the 2008/2009 survey. However, the authors claimed that any comparisons between surveys should be treated with caution as there were significant differences with regard to sample sizes, as well as the number of sessions per school (Ipsos MORI 2014).

Finally, another representative dataset concerning adolescent gambling was derived from the 2011 ESPAD study (Molinaro et al. 2014). The sample comprised 1712 students from the whole UK and the rate of probable problem gambling according to Lie/Bet Scale was 2.2 %.

#### Greece

There have been very few studies on adolescent gambling behaviour in Greece. To our knowledge, only one study (conducted in the Greek island of Kos) examining adolescent Internet gambling (Floros et al. 2013) has been conducted. In this study, the sample comprised 2017 students between 12 and 19 years of age. The DSM-IV-MR-J was used to assess problematic Internet gambling, and the Parental Bonding Instrument (PBI) was used to assess parental care and overprotection. The results indicated that 37.2 % of adolescents reported having had some experience with Internet gambling, whereas 4.1 % of the total participants were classified as problem gamblers (Floros et al. 2013). Parental care was associated with lower scores on the DSM-IVMR-J, whereas higher levels of overprotection were associated with higher scores (Floros et al. 2013).

#### Iceland

With regard to adolescent gambling, a study was conducted in Iceland in 2003 that included a sample of 750 students aged 16–18 years (Olason et al. 2006a). Icelandic versions of DSM-IV-MR-J and SOGS-RA were administered to students during class time. The most popular gambling activities in the 12 months prior to the survey were scratch tickets (53.7 %), gambling machines (46.7 %) and Lotto (30.4 %). With regard to problem gambling, the DSM-IVMR-J identified 2.0 % of the sample as problem gamblers and 3.2 % as at-risk problem gamblers. The SOGS-RA identified 2.7 % as problem gamblers with a further 4.4 % at risk for developing gambling problems. According to both instruments, females were less likely to be classed as problem gamblers.

Another study was conducted with 3511 adolescents aged 13–15 years in schools in Reykjavík (Olason et al. 2006b). The most popular gambling activities in the 12 months prior to the survey were scratch tickets (48.2 %), Electronic Gaming Machines (EGMs) (32.0 %) and Lotto (28.1 %). With regard to problem gambling, the DSM-IV-MR-J identified 1.9 % as problem gamblers with 3.7 % as at-risk problem gamblers, and 8 % had gambled at least once a week for the preceding 12 months. The SOGS-RA identified 2.8 % as problem gamblers with a further 4.1 % at risk for gambling problems. Boys had more gambling problems than girls. Further analysis showed that problem and at-risk gamblers were more likely than other gambling groups to report that their parents gambled (10 % of problem gamblers and 5 % of at-risk gamblers compared to only 2 % of the social gamblers that reported that their parents gambled).

The most recent adolescent survey was conducted in the academic year 2007–2008, in which 1537 students aged 13–18 years participated (Olason et al. 2011), and the DSM-IV-MR-J was used to assess problem gambling. The most popular past year gambling activities were scratch tickets (27.1 %), poker (22.3 %) and EGMs (21.4 %). With regard to Internet gambling, 24.3 % reported wagering money and 4.1 % did it at least on a weekly basis. The most popular forms of gambling on the Internet were casino type games on non-Icelandic websites (12.4 %), Internet Lotto (8.7 %) and sport pools (8.5 %). The findings showed that 2.7 % of the participants were at-risk gamblers and 2.2 % were problem gamblers. Problem gambling was more prevalent among boys (4.2 % compared to 0.1 % of females) and among adolescents aged 15–16 years (4.2 %) compared to 13–14 years (0.7 %) and 17–18 years (2.2 %). In addition, the prevalence of problem gambling among Internet gamblers (7.5 %) was considerably higher than that obtained for the total sample (2.2 %).

#### Italy

Youth gambling in Italy has been examined via the Italian Population Survey on Alcohol and Drugs (IPSAD) 2007–2008 study, and comprises a sub-sample of individuals aged from 15 to 24 years old (Bastiani et al. 2011). The findings showed that Italian youth, although gambling less than adults (35.7 vs. 45.3 %), appear to have a higher prevalence of moderate risk or problem gambling (2.3 vs. 2.2 %). Among this age group, male gender, primary educational level, and smoking more than 10 cigarettes per day were more associated with moderate-risk and problem gambling (Bastiani et al. 2011).

Another representative study of Italian adolescent gambling behaviour was derived from the 2011 ESPAD study (Molinaro et al. 2014). In this study, the Italian sample comprised 4837 participants and the rate of probable problematic gambling was 2.6 % using the Lie/Bet Scale.

#### Lithuania

A Lithuanian study on adolescent problem gambling behaviour was carried out in Kaunas, with 835 adolescents, aged 10–18 years (Skokauskas and Satkeviciute 2007). Problem gambling was assessed via two different screens (i.e., SOGS-RA and DSM-IV-MR-J). The results showed that the most popular gambling activities among all gamblers was Tele-Lotto (53.9 %) followed by other lotteries (36.8 %) and betting (9.8 %). Based on the DSM-IV-MR-J, 4.2 % of respondents were classed as problem gamblers, 9.1 % classed as at-risk gamblers, 69.4 % were classed as social gamblers, and 17.3 % as non-gamblers. With respect to the reasons for gambling, the largest proportion of participants reported that gambling was mainly a form of enjoyment (49.1 %), followed by the reasons “a chance to try luck” (44.6 %), and “to win money” (34.1 %). In fact, the reasons for gambling varied according to the severity of gambling (i.e., the reasons for winning money were less referred to by problem gamblers). Adolescent problem gamblers in comparison with non-problem gamblers were more likely to engage on slot machines (51.4 vs. 8.1 %) and cards (17.1 vs. 6.9 %). Problem gamblers were found to be older (mean = 16.6 years), than at-risk (mean = 15 years) and social gamblers (mean = 14.5 years). Moreover, problem gamblers were more likely to be male (6.3 %) than female (2.3 %), and were significantly more likely to report that their parents gambled (80.1 %), compared to social gamblers (12.8 %). Furthermore, problem gamblers were more likely to engage in regular alcohol use (57.1 %) than social gamblers (10.2 %); and also more likely be involved in cigarette smoking (65.7 %) than social gamblers (12.7 %). Finally, 2.9 % of problem gamblers reported using illegal drugs regularly as compared to 1.3 % of social gamblers.

There are also data about adolescent gambling in Lithuania derived from the 2011 ESPAD study (Molinaro et al. 2014). The sample comprised 2476 participants and the rate of probable problem gambling using the Lie/Bet Scale was 4.2 %.

#### Luxembourg

A survey of gambling behaviours among high-school students was carried out by the Luxembourg Centre of Addiction Prevention (Centre de Prévention des Toxicomanies). The study did not assess problem gambling among the students, but examined the most common gambling activities, and how frequently youth engaged in each form of gambling (Duscherer and Paulos 2014). Data from 3496 students indicated that the most frequent gambling activities cited by youth were scratchcards (71 %), card games (58.4 %) and slot machines (51.8 %). Apart from slot machine gambling where there were no gender differences (51.7 % males vs. 52 % females), boys engaged more frequently in all gambling activities (Duscherer and Paulos 2014). However, the authors hypothesized that this small difference between males and females for slot machines may have been due to the wording used in the German survey, as the German word “Spielautomat” may have been interpreted as a video game device (Duscherer and Paulos 2014).

#### Norway

In assessing adolescent gambling, some Norwegian school surveys have been carried out. The first survey was carried out by Johansson and Götestam (2003) and comprised 3237 participants aged 12–18 years. The results also showed that 1.76 % of participants were pathological gamblers as assessed using the DSM-IV, and 3.46 % were at-risk gamblers (Johansson and Götestam 2003). Males were more likely to be classified as pathological gamblers than females (2.79 % males vs. 0.69 % females) (Johansson and Götestam 2003).

Another youth survey was conducted, with a sample of 1351 students aged 16–19 years (Molde et al. 2009). The instrument used to assess problem gambling was the DSM-IV subscale of the Massachusetts Adolescent Gambling Screen (MAGS). The prevalence of problem gambling was 1.9 % (score 3–4.5) and the prevalence of pathological gambling was 2.5 % (score 5+). The combined prevalence of problem and pathological gambling was 4.4 %. A multiple logistic regression analysis showed that gender, depression, alcohol abuse, and dissociation were related to problem and pathological gambling.

In September 2004, an adolescent survey was carried out with students aged 13–19 years from all geographical regions in Norway, during regular school classes (Rossow and Molde 2006). The sample comprised 20,703 participants and the Lie/Bet Scale and the SOGS-RA were used to assess problem gambling (Rossow and Molde 2006). The most reported gambling activities were scratch lottery tickets (55.5 %) and slot machines (50.2 %). The problem rate was 2.5 % using the SOGS-RA and 3.5 % using the Lie/Bet Scale (score 2). Problem gambling prevalence rates were higher among boys, among those who did not live with both parents, and among those with an Islamic or other non-Christian religious affiliation (Rossow and Molde 2006).

Another survey before (2006) and after (2008) the removal of slot machines was designed to assess the possible impact of this intervention (Rossow et al. 2013). The analysis was carried out only in students from the same schools in both survey years. The students who participated were aged between 13 and 18 years. The samples comprised 4912 students in 2006 and 3855 students in 2008. To assess problem gambling, three instruments were used: SOGS, Lie/Bet Scale, and a single question about self-perceived gambling problems – “Do you think that you have problems due to your gambling?” When comparing the prevalence of gambling behaviour reported by respondents in 2006 and 2008, the weekly prevalence of the majority of gambling activities decreased from 2006 to 2008, except on internet poker (3.0 % in 2006 and 3.8 % in 2008). With regard to the prevalence of at-risk and problem gambling between 2006 and 2008, there was a statistically significant decrease in the prevalence of self-perceived gambling problems (3.6 % in 2006 and 2.3 % in 2008). However, there was no statistically significant difference in the prevalence of the Lie/Bet Scale and there was a statistically significantly higher prevalence of SOGS-RA in 2006 compared to 2008 (2.3 % in 2006 and 3.1 % in 2008).

More recently, a prevalence survey with 2055 Norwegian adolescents aged 17 years reported a problem gambling prevalence of 0.2 % using the PGSI (Hanss et al. 2014). Here, being male, living without both parents, and being of non-Norwegian ethnicity, were more associated with problem gambling.

#### Romania

The first Romanian study conducted by Lupu et al. (2002) was carried out among 500 school teenagers from three different Romanian districts (Cluj, Salaj and Bacau) and the GA-20 was used to assess problem gambling. The findings of this study indicated that 6.8 % of adolescents were problem gamblers, with males being more likely to be problem gamblers (82.4 %) than females (17.6 %). In addition, the most frequent gambling activities were pool betting (55.9 %), poker (35.3 %), and bingo (32.4 %).

The most recent adolescent gambling study was carried out by Lupu and Todirita (2013), with 1032 adolescents aged 11–19 years. The GA-20 was again used to assess problem gambling. The results showed that 73 % gambled at a recreational level and 3.5 % at a pathological level. Males were more likely to be pathological gamblers than females. The mean age of pathological gamblers was 16.5 years (Lupu and Todirita 2013). The games most played by pathological gamblers were sports betting/slot machines (36 % of players) and lotto/internet casino/pool bets (25 %). Moreover, pathological gambling was associated with alcohol (66.7 %), illegal drugs (13.9 %), legal drugs (19.4 %), and smoking cigarettes (16.7 %).

Another representative dataset concerning adolescent gambling was derived from the 2011 ESPAD study (Molinaro et al. 2014). The Romanian sample comprised 2770 students aged 16 years and the rate of probable problem gambling was 4.9 % using the Lie/Bet Scale.

#### Serbia

There are some data available for this country, derived from the 2011 ESPAD Study (Molinaro et al. 2014). The Serbian sample comprised 6084 participants and the probable problem gambling rate was 3.1 % (scoring 2 on the Lie/Bet Scale).

#### Spain

A study carried out by Becoña et al. (2001) was conducted with a representative sample of 2790 students from Galicia aged between 14 and 21 years. The SOGS-RA was used to assess problem gambling. The findings of this study showed that 86.1 % were non-problem gamblers, whereas 8.2 % were at-risk gamblers, and 5.6 % were problem gamblers during the past year. Within the group of problem gamblers, 10.4 % were boys and 1.6 % were girls. With regard to age, 6 % of problem gamblers had between 14 and 17 years and 4.6 % of problem gamblers were aged between 18 and 21 years old. In addition, 12.7 % of problem gamblers had parents who gambled excessively, whereas only 1.6 % of non-gamblers had parents who gambled excessively (Becoña et al. 2001).

A more recent study was carried out by Míguez and Becoña (2015), with 1447 students from Galicia (aged 11–16 years), who completed an anonymous survey in the class. The study found a problem gambling rate of 4.6 % using the SOGS-RA. This study also showed a clear relationship between a greater involvement in gambling, cigarette smoking, and alcohol use.

#### Sweden

Fröberg et al. (2015) conducted a study, using nationally representative data, in order to estimate the incidence of a first episode of problem gambling among Swedish youth. Participants were first interviewed (from October 2008 to April 2009), and in a second wave (December 2009 to May 2010) participants were approached for a follow-up interview. At baseline, participants were distributed in three groups: baseline problem gamblers (defined as having a score of 3–27 in the PGSI in the past 12 months); baseline previous problem gamblers (defined as participants without problem gambling in the past 12 months, but with problem gambling previously in life measured by the SOGS-R); and baseline never problem gamblers (participants who never had a gambling problem, that is, participants with a score of 0–2 in the PGSI and in the SOGS-R). Among 2318 of youth aged 16–24 years, who were baseline never problem gamblers, 2.3 % had a first episode of problem gambling measured by the PGSI at the follow-up. Furthermore, the incidence proportion of a first episode of problem gambling was higher among males (3.2 %) than females (1.1 %), as well as among individuals who were born outside of Sweden (4.3 % for those born in Europe and 3.7 % for those born elsewhere vs. 2.1 % for those who were born in Sweden). In addition, the prevalence of problem gambling at the follow-up among all individuals aged 16–24 years (and not only among those who were never baseline problem gamblers) was 4.2 %.

#### Switzerland

A youth gambling prevalence study was conducted using data drawn from the 2007 Swiss Health Survey (Luder et al. 2010), with participants aged 15–24 years. Data on gambling behaviour were available for 1116 students. These students were surveyed about gambling frequency, but no standardized measures of problem gambling were used in this study. The results showed that 48.3 % of youth were engaged in some gambling activity during the previous year, whereas 34.8 % were classified as occasional gamblers (gambled < 52 times/year) and 13.5 % were classified as frequent gamblers (gambled > 52 times/year). In addition, frequent gamblers had higher odds of being daily cigarette smokers.

A regional study that also assessed adolescent problem gambling was conducted in the canton of Neuchatel (Surís et al. 2011). This study comprised a sample of 1126 students aged 15–20 years, who participated in an online survey. The French version of the SOGS-RA was used to assess problem gambling. According to this study, 31.9 % were non-problem gamblers, whereas 4.3 % were at-risk gamblers, and 1.3 % were problem gamblers. The group of at risk/problem gamblers was predominantly boys (82 %). The proportion of these gamblers increased with age and decreased with socioeconomic level. The types of gambling activities most engaged by youth were lotteries (80.2 %), gambling on casinos and on the Internet (55.5 %). In addition, the results showed a significant association between being at-risk/problem gamblers and the problematic use of Internet, as well as the use of psychoactive substances, such as tobacco, alcohol, cannabis and other illegal drugs.

## Discussion

Several conclusions can be drawn from this review of adolescent prevalence studies. Firstly, from a methodological perspective, school-based surveys were the primary modality to collect data for adolescent prevalence surveys. However, all the data are self-report, and are subject to many well-known biases, such as the reliability of memory, social desirability and the honesty of the responses given.

Secondly, some variations in the sample sizes can be found. Moreover, some studies used local or regional samples, whereas other studies used national samples, which allow for a better generalizability to the general population.

Thirdly, some instruments used a past-year timeframe, whereas others used a lifetime perspective. Logically, a lifetime perspective will produce higher problem gambling prevalence rates. Fourthly, it should be noted that different studies used different screening instruments to assess problem gambling, which makes comparing prevalence rates across countries more difficult. In addition, the majority of gambling instruments, despite being designed for adolescents, are developments of gambling screens that were originally designed to assess gambling problems among adults, with wording and content adapted to be more suitable for adolescent populations. Only the study conducted in Croatia (i.e., Dodig 2013) used the CAGI, an instrument originally developed for use with adolescents. However, pending a better-validated problem gambling instrument for adolescents, these instruments are likely to continue to be viewed as the best approximations for the assessment of problem gambling among adolescents.

The lifetime worldwide problem gambling prevalence rates ranged from 1.6 to 5.6 %. The lowest problem gambling rates were found in Brazil and Denmark and the highest was found in Albania. The low prevalence rates found in Brazil and Denmark could be related to the gambling legislation and access to gambling venues in these countries. Looking at the European continent, the lifetime problem gambling prevalence rates ranged from 1.6 to 5.6 %, with Albania again reporting the highest problem gambling prevalence rate (see Molinaro et al. 2014). The highest rate found in Albania could be related to the effect of living in Eastern and Balkan countries, where welfare services and health benefits are limited, and which may have a negative impact on adolescent health (Holstein et al. 2009). Such a finding suggests that more research on youth gambling should be carried out in this country, in order to examine the specific factors in this particular context that may influence gambling and problem gambling behaviour.

The past-year adolescent gambling prevalence rates across the world ranged from 0.2 to 5.6 %, with Spain presenting the highest prevalence rate (although this rate was not found in the most recent adolescent study of this country). The lowest prevalence rate of 0.2 % was found in Australia and Norway. The low prevalence rate found in the Australian study conducted in the Darwin metropolitan area might be due to a selective under-sampling of young people with lower school attendance or levels of literacy, as suggested by the authors (i.e., Delfabbro and King 2011).

In Europe, the adolescent gambling prevalence rates ranged from 0.2 (in Norway) to 5.6 % (Spain). The high prevalence rate in Spain may be due to the existence of different types of slot machines that were easily accessible in the country at the time the study was conducted (see Becoña et al. 2001). However, all the Spanish studies are regional and therefore national studies should be conducted. The lowest rate found in Norway could be due to a possible effect of recent changes in the gambling landscape, such as the removal of slot machines in 2007 and the reintroduction of new slot machines with less addictive potential in the Autumn of 2008 (see Rossow et al. 2013).

Another noteworthy aspect is a high prevalence rate found in Croatia (12.3 %) with a timeframe of 3-months. This finding might be due to the instrument used (i.e., CAGI), which has not been used in any other study. It may also be explained by the easy accessibility of gambling in Croatia (see Dodig 2013) and highlights the need to conduct more research in this country with instruments used in other studies.

Overall, these findings suggest some variations in youth problem gambling prevalence rates across different countries and show the need to encourage more collaboration between researchers from different countries using the same instrumentation and employing the same cut-offs, in order to improve comparability between national studies, and to understand the effect of different legislation in youth gambling patterns.

Notwithstanding these variations, there were also consistent results in demographics: many European countries showed that adolescent problem gambling is more likely to occur among males (Johansson and Götestam 2003; Ashworth and Doyle 2000; Griffiths and Wood 2007; Skokauskas and Satkeviciute 2007; Kristiansen and Jensen 2014; Dodig 2013; Raisamo et al. 2013; Surís et al. 2011; Hanss et al. 2014; Fröberg et al. 2015; Bastiani et al. 2011; Becoña et al. 2001; Olason et al. 2006a, b; Olason et al. 2011); among those individuals who belong to an ethnic minority (Hanss et al. 2014; Fröberg et al. 2015; Forrest and McHale 2012; Griffiths 2008); among those who have parents who gambled (Becoña et al. 2001; Forrest and McHale 2012; Olason et al. 2006b); and who did not live with both parents (Rossow and Molde 2006; Hanss et al. 2014); and among older adolescents (Skokauskas and Satkeviciute 2007; Surís et al. 2011; Kristiansen and Jensen 2014). Problem gamblers were also more likely to gamble on the Internet (Olason et al. 2011; Kristiansen and Jensen 2014), which could be explained by some characteristics of this gambling mode, such as its accessibility, affordability, convenience and anonymity. In fact, Internet gambling may serve as a good modality for young people engage in gambling activities without age verifications and parental supervision, and therefore may explain the vulnerability of this age group to modern and remote forms of gambling, as some authors had already suggested (e.g., Delfabbro et al. 2009).

Overall, the most frequent motivations reported by adolescent problem gamblers were gambling to escape and the inability to resist temptation (Skokauskas and Satkeviciute 2007; Kristiansen and Jensen 2014). A noteworthy aspect was that gambling for winning money was a motivation less mentioned by adolescent problem gamblers in comparison with non-problematic adolescent gamblers. This finding may be understood in light of existing gambling theories, namely the need-state and dispositional theories of gambling (see Griffiths and Delfabbro 2001), which claims that people gamble in order to escape from problems and unpleasant feelings, and that problem gambling can be viewed as a maladaptive coping strategy used to handle stress and/or depression (Blaszczynski and McConaghy 1989). In fact, according to this theory, the excitement of gambling could also be independent of people’s desire to win money (Walker 1992). Significant predictors of problem gambling include the experience of winning a large amount of money early in adolescents’ playing career, and being of lower socioeconomic status (Dodig 2013; Surís et al. 2011).

Furthermore, most empirical research on adolescent gambling in Europe has demonstrated a clear relationship between gambling behaviour and substance abuse (Molde et al. 2009; Luder et al. 2010; Bastiani et al. 2011; Míguez and Becoña 2015; Lupu and Todirita 2013; Skokauskas and Satkeviciute 2007; Griffiths and Wood 2007). This finding suggests that gambling is maintained by many of the same processes inherent in these other addictions, which may lend some support to the theory that gambling is also a biopsychosocial addiction, like alcoholism and substance dependence (Walker 1992; Griffiths 1995). However, as other researchers have noted, it should borne in mind that although there are some biologically-related factors that can account for gambling behaviour (e.g., tolerance, withdraw and craving), these factors fail to explain some of the other findings observed in the present review, for example, the variations in motivations to gamble and why some gambling activities appear to be more “addictive than others” (Griffiths and Delfabbro 2001).

In fact, the most popular youth gambling activities tended to be lotteries, scratchcards, card games, and slot machines (Duscherer and Paulos 2014; Rossow and Molde 2006; Ashworth and Doyle 2000; Olason et al. 2006a, b; Olason et al. 2011; Griffiths 2008; Softić et al. 2013). In addition, problem gamblers participated in more different gambling activities than non-problem gamblers (Kristiansen and Jensen 2014). The games most played by problem gamblers were slot machines (Forrest and McHale 2012), card games, and sports betting (Skokauskas and Satkeviciute 2007; Lupu and Todirita 2013). The gambling activities that appeared to be the most problematic included those that involve high event frequencies and short interval between stake and payout (such as slot machines), and confirms previous studies that have identified slot machines as one of the most problematic types of gambling worldwide (Abbott 2001; Parke and Griffiths 2006). The structural characteristics of slot machines, such as the high event frequencies, immediacy of rewards, and short interval between stake and payout, might facilitate the maintenance of the behaviour by means of operant conditioning (Griffiths and Delfabbro 2001). In addition, such machines are highly accessible and available in many European countries, as they are found in the majority of bars, restaurants and amusement arcades, and can be played with relatively little money (Chóliz 2010).

Overall, the present review supports the findings of other reviews with regard to demographics, and behavioural characteristics that are more associated with youth gambling involvement. In addition, other reviews also highlight the fact that different methodological procedures and different instruments with different cut-offs can generate differences in problem gambling prevalence rates between studies (e.g., Kristiansen and Jensen 2011; Volberg et al. 2010). Notwithstanding these similarities, this review expands and updates the previous reviews, which are now outdated, and tried to overcome the English publication bias by providing youth problem gambling prevalence rates of some countries which are only available in their native languages (e.g., Spain, Switzerland). Moreover, unlike other reviews, the present review provided a country-by-country analysis of adolescent gambling across a whole continent (i.e., Europe). This goal is particularly relevant because such an overview can encourage the development of a more common European prevention strategy that could reach a larger number of young people.

It has also been noted by other researchers that youth show higher problem gambling prevalence rates than adults (e.g., Shaffer and Hall 2001). Furthermore, in comparison with other systematic reviews on adult gambling prevalence studies (e.g., Stucki and Rihs-Middel 2007), the problem gambling prevalence rates among youth using a past-year (or shorter timeframe) are higher. These observed differences in problem gambling rates between young people and adults have multiple explanations. Firstly, adolescence is a transitional period already characterized by risk taking behaviours more generally (Arnett 1992). Consequently, most adolescents decrease their risk-taking behaviour (including gambling), as they mature into adulthood and assume adult roles, such as having a full time job, getting married, or having a child (Griffiths 1995). Secondly, the current generation of youth is the first to grow up in a society where gambling is generally accepted, heavily available, and widely promoted (Volberg et al. 2010). This might facilitate youth engagement in this behaviour without a careful examination of its risks and hazards. The fact that adolescents and young adults constitute a vulnerable age group for the development of gambling-related problems has many implications, as youth gambling has many social costs (Gupta and Derevensky 2014). This will hopefully stimulate more research among underage youth, especially longitudinal designs in order to examine the determinants of problem gambling and how youth gambling behaviour can change over time. In addition, this review should hopefully (1) encourage policymakers to think about more effective legislation to protect this vulnerable group and (2) help practitioners dealing with young people to be aware that gambling is a behaviour that might be part of a more global risky behaviour lifestyle.
